# Decreased TPD52 expression is associated with poor prognosis in primary hepatocellular carcinoma

**DOI:** 10.18632/oncotarget.6319

**Published:** 2015-11-13

**Authors:** Ying Wang, Chang-Long Chen, Qiu-Zhong Pan, Ying-Yuan Wu, Jing-Jing Zhao, Shan-Shan Jiang, Jie Chao, Xiao-Fei Zhang, Hong-Xia Zhang, Zi-Qi Zhou, Yan Tang, Xu-Qiong Huang, Jian-Hua Zhang, Jian-Chuan Xia

**Affiliations:** ^1^ Collaborative Innovation Center for Cancer Medicine, State Key Laboratory of Oncology in South China, Sun Yat-Sen University Cancer Center, Guangzhou, China; ^2^ Department of Health Service Management, Guangzhou University of Chinese Medicine, Guangzhou, China; ^3^ Department of Epidemiology and Health Statistics, Guangdong Key Laboratory of Molecular Epidemiology, Guangdong Pharmaceutical University, Guangzhou, China; ^4^ Department of Gynaecology and Obstetrics, Panyu Branch of Armed Police Corps Hospital of Guangdong, Guangzhou, China

**Keywords:** TPD52, hepatocellular carcinoma, prognosis, tumor suppressor, mechanism

## Abstract

Tumor protein D52 (TPD52) has been indicated to be involved in tumorigenesis of various malignancies. But its role in hepatocellular carcinoma (HCC) is unknown. This study aimed to explore the expression of TPD52 in HCC samples and cell lines using real-time quantitative PCR, western blotting, and immunohistochemistry. The prognostic value of TPD52 in HCC was also analysed. Meanwhile, the mechanism of TPD52 in hepatocarcinogenesis was further investigated by western blotting, immunohistochemistry, over-express and knockdown studies. We found that TPD52 expression was significantly decreased in the HCC tissues and HCC cell lines. TPD52 expression was significantly correlated with tumor-nodes-metastasis (TNM) stage. Kaplan–Meier survival curves showed that high TPD52 expression was associated with improved overall survival (OS) and disease-free survival (DFS) in HCC patients. Multivariate analysis indicated that TPD52 expression was an independent prognostic marker for the OS and DFS of patients. In addition, TPD52 expression was positively correlated with p21 and p53 expression, and was negatively correlated with MDM2, BCL2 and P-GSK-3β expression in HCC. In conclusions, our findings suggested that TPD52 is a potential tumor suppressor in HCC. It may be a novel prognostic biomarker and molecular therapy target for HCC.

## INTRODUCTION

Hepatocellular carcinoma (HCC) is the fifth most prevalent neoplasm and the third most frequent cause of cancer-related death [1]. Most HCC cases (80%) are in eastern Asia and sub-Saharan Africa, where the main risk factor is chronic hepatitis B virus (HBV) infection, together with exposure to aflatoxin B1 [2]. Currently, surgical resection is the first-line treatment for HCC; however, it is usually limited by many factors, such as liver dysfunction, patient condition, and multifocality [3, 4]. Therefore, this has given rise to various alternative treatments for HCC, including percutaneous ethanol injection, radiofrequency ablation therapy, and transarterial chemoembolization [5]. As HCC is not sensitive to conventional chemotherapy or radiotherapy, adjuvant immunotherapy might benefit patients with HCC who undergo resection, as any residual tumor would probably be minimal. Unfortunately, intrahepatic recurrence is common; therefore, the prognosis of HCC remains poor even with aggressive therapies, where the 5-year survival rate is as low as 25%-39% [6]. To improve outcomes, there is an urgent need to identify novel and efficient new targets for early diagnosis and effective treatment of HCC [7].

Hepatocarcinogenesis is a complex multistep process that involves activating oncogenes and inactivating tumor suppressor genes, in which many signaling cascades are altered and lead to a heterogeneous molecular profile [8-10]. Genetic factors, such as loss of heterozygosity, microsatellites, chromosome instability, and hypermethylation, have been reported in association with HCC [11]. Investigating and clarifying the roles of the genes involved in HCC development will contribute to our understanding of the mechanisms of hepatocarcinogenesis [7]. It is also significant for improving HCC diagnosis and treatment as well as predicting prognosis.

The tumor protein D52 (*TPD52*) gene was identified about 20 years ago. It is located at chromosome 8q21, at a region that is frequently gained or amplified in multiple human cancers [12-14]. Besides *TPD52*, this mammalian gene family contains three other genes: *TPD52L1*, *TPD52L2*, and *TPD52L3* [15]. Human TPD52 isoforms are 200 amino acid residues in length and contain a number of sequence motifs, such as a coiled-coil motif, and N- and C-terminal-located proline, glutamic acid, serine, and threonine (PEST) sequences[12]. Numerous studies have revealed that TPD52 is involved in regulating cell survival, proliferation, migration, and invasion, DNA repair, exocytosis, and vesicle trafficking [16-22]. However, its roles in cancer are controversial. TPD52 is overexpressed in several cancers, such as ovarian, breast, prostate, and pancreatic cancer, and multiple myeloma, Burkitt's lymphoma, and melanoma [23-29]. TPD52 is also down-regulated in certain tumors, such as papillary renal cell cancer, leiomyosarcoma, clear cell renal cell cancer, liposarcoma, and lung cancer [30]. Although TPD52 has been investigated in several cancers, to our knowledge, there are no reports on its expression and prognostic value in HCC.

In this study, we investigated the expression of TPD52 in primary HCC using real-time quantitative reverse transcription-PCR, western blotting, and immunohistochemistry. Additionally, we evaluated the relationship between TPD52 expression and the clinicopathological features of HCC, and investigated the prognostic value of TPD52 in HCC. The mechanism of TPD52 in hepatocarcinogenesis was also investigated.

## RESULTS

### TPD52 mRNA and protein expression in primary HCC tissue samples and HCC cell lines

For the detection of TPD52 mRNA expression, 1 μg of total RNA were needed to perform the reverse transcription. For the detection of TPD52 protein expression, about 27 μg of protein were needed. However, in the 40 paired samples collected from HCC patients, some samples (cancerous tissues, or adjacent noncancerous tissues) were very small, and the RNA and protein may be degrading during the storage. Some samples were not enough to extract sufficient RNA and protein. So we chose 33 paired and 25 paired samples from the 40 paired samples to perform real-time PCR and western-blot analysis, respectively.

**Figure 1:**
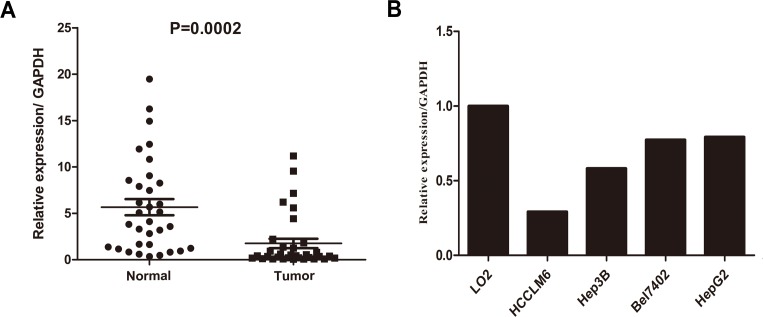
Real-time quantitative PCR evaluation of *TPD52* mRNA expression in primary HCC surgical specimens and HCC cell lines **A.** The relative *TPD52* mRNA expression was significantly lower in 33 tumor tissues than in the matched adjacent non-tumor tissues (*P* = 0.0002). **B.** Compared with the normal liver cell line LO2, *TPD52* mRNA expression was down-regulated in the HCCLM6, Hep3B, Bel7402, and HepG2 HCC cells.

Real-time quantitative PCR was performed on 33 paired clinical samples from patients with HCC (tumor tissues and matched adjacent non-tumor liver tissues) and HCC cell lines to determine their *TPD52* mRNA levels. *TPD52* mRNA expression was significantly down-regulated in 28/33 (85%) tumor tissues as compared with the matched adjacent non-tumor tissues (*P* = 0.0002, Figure 1A). Furthermore, TPD52 transcript levels were decreased in the HepG2, Hep3B, HCCLM6, and Bel7402 HCC cell lines relative to the LO2 normal liver cell line (Figure 1B).

**Figure 2:**
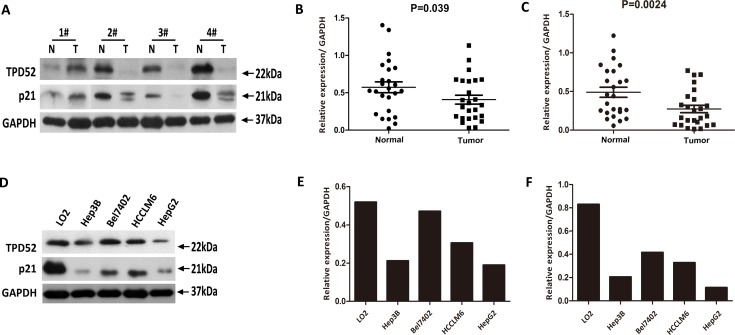
Western blotting evaluation of TPD52 and p21 protein expression in primary HCC surgical specimens and HCC cell lines **A.** Representative result of expression of TPD52 and p21 protein in the same four pairs HCC tissues and matched adjacent non-cancerous tissues (N, matched non-cancerous tissues; T, HCC tissues). **B.** Relative expression of TPD52 protein was lower in tumor tissues than in the matched adjacent non-tumor tissues (*P* = 0.039, *n* = 25). **C.** Relative expression of p21 protein was lower in tumor tissues than in the matched adjacent non-tumor tissues (*P* = 0.0024, *n* = 25). **D.** Result of expression of TPD52 and p21 protein in the same HCC cell lines and the normal LO2 liver cell line. **E.** TPD52 protein level was significantly lower in the Bel7402, HCCLM6, Hep3B, and HepG2 cells than in LO2 cell. **F.** p21 protein level was also significantly lower in the Bel7402, HCCLM6, Hep3B, and HepG2 cells than in LO2 cell.

TPD52 protein level was also detected on 25 paired fresh HCC tissues and matched control tissues,and HCC cell lines by western blotting analysis. Consistent with the real-time quantitative PCR results, TPD52 protein expression was decreased in 17/25 (68%) tumor tissues (*P* = 0.039, Figure 2A and 2B). Likewise, compared to the LO2 cells, TPD52 protein expression was decreased in the HCC cells, especially in the Hep3B and HepG2 cells (Figure 2D and 2E).

### Immunohistochemical analysis of TPD52 expression in HCC clinical samples and its relationship to clinicopathological parameters

**Figure 3:**
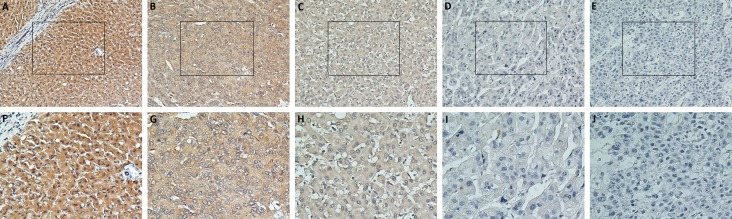
Immunohistochemical analysis of TPD52 protein expression in primary HCC surgical specimens **A.** and **F.** Strongly stained normal liver tissue distant from the tumor. **B.** and **G.** Well-stained tumor tissues (TPD52+++). **C.** and **H.** Moderately stained tumor tissues (TPD52++). **D.** and **I.** Weakly stained tumor tissues (TPD52+). **E.** and **J.** Negatively stained tumor tissues (TPD52-). (A-E,×200 magnification; F-J, ×400 magnification).

TPD52 expression was investigated in 154 HCC surgical specimens using immunohistochemical staining. TPD52 was detected in the cytoplasm of the positive-stained cells (Figure 3). 68 cases (44.1%) had high TPD52 expression (TPD52+++ or TPD52++); the remaining 86 cases (55.9%) had low TPD52 expression (TPD52+ or TPD52-) (Table 1). Table 1 lists the relationship between TPD52 expression and the clinicopathological parameters. The correlation analysis suggested that TPD52 expression was significantly correlated with tumor-nodes- metastasis (TNM) stage (*P* = 0.011).

**Table 1:** Relationship between TPD52 expression and clinicopathological features of patients with HCC (*n* = 154)

Clinicopathologic variables	Number	TPD52 expression		*P* value
		low	high	
All cases	154	86	68	
Age (years)				0.623
<50	89	51	38	
≥50	65	35	30	
Gender				0.842
Male	123	70	53	
Female	31	16	15	
HBV				0.325
Negative	21	14	7	
Positive	133	72	61	
Tumor size(cm)				0.745
<5	77	42	35	
≥5	77	44	33	
Tumor number				0.838
Single	124	70	54	
Mutiple	30	16	14	
Liver cirrhosis				1.000
No	33	19	14	
Yes	121	67	54	
Tumor encapsulation				0.662
None	58	33	25	
Complete	53	31	22	
Incomplete	43	22	21	
Serum AFP				0.245
<400	95	49	46	
≥400	59	37	22	
Histilogical differentiation				0.178
Well	52	26	26	
Moderate	66	35	31	
Poor	36	25	11	
TNM stage				0.011*
I-II	100	48	52	
III	54	38	16	

AFP, alpha-fetoprotein.

* Statistically significant (*P* < 0.05).

### Relationship between TPD52 expression and survival

**Figure 4:**
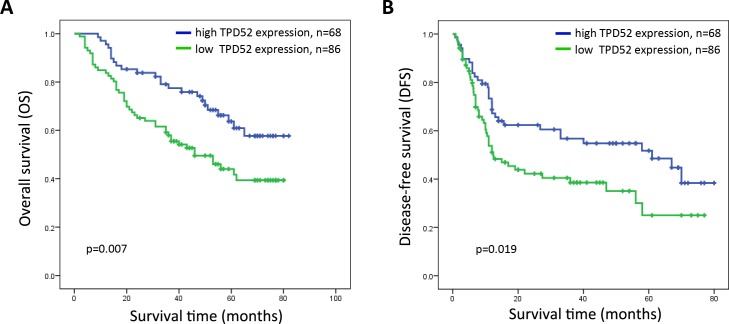
Kaplan-Meier survival curves of patients with primary HCC after surgical resection according to the expression of TPD52 **A.** The overall survival rate of patients in the low-TPD52 group was significantly lower than that of patients in the high-TPD52 group (*P* = 0.007). **B.** The disease-free survival rate of patients in the low-TPD52 group was also significantly lower than that of patients in the high-TPD52 group (*p* = 0.019).

The prognostic value of TPD52 for survival was evaluated by comparing high and low TPD52 expression in the patients with HCC. The median OS and DFS for patients with high TPD52 expression were 51 and 26 months compared with 38 and 11months for patients with low TPD52 expression, respectively. The OS rates at 1-, 3-, and 5-years were 95.6%, 77.5%, and 63.7% for patients with high TPD52 expression compared to 83.7%, 58%, and 44% for patients with low TPD52 expression (log-rank test, *P* = 0.007). The DFS rates at 1-, 3-, and 5-years were 68.7%, 56.7%, and 51.7%, for patients with high TPD52 expression compared to 51.1%, 38.5%, and 25% for patients with low TPD52 expression (log-rank test, *P* = 0.019) (Figure 4). These results indicated that low TPD52 expression was significantly associated with poor prognosis in HCC.

Univariate and multivariate analyses were carried out to examine the effect of TPD52 expression on HCC prognosis using a Cox proportional hazard model. TPD52 expression showed a significant correlation with improved OS and DFS of patients in univariate analysis (Table 2 and Table 3). Multivariate Cox regression analysis further indicated that TPD52 expression was an independent predictor of OS and DFS (Table 2 and Table 3). Accordingly, TPD52 expression may be useful for predicting survival in HCC.

**Table 2:** Univariate and multivariate analysis of overall survival in HCC

Variables	Univariate analysis			Multivariate analysis		
	HR	95% CI	p value	HR	95% CI	*P* value
Age	0.780	0.507-1.201	0.259			
Gender	1.050	0.625-1.764	0.853			
HBV	1.250	0.646-2.417	0.508			
Tumor size	1.527	1.003-2.324	0.048*	1.340	0.873-2.058	0.181
Tumor number	1.731	1.067-2.811	0.026*	1.627	0.997-2.654	0.051
Tumor encapsulation	1.161	0.888-1.517	0.276			
TNM stage	2.210	1.446-3.678	<0.001*	1.708	1.085-2.689	0.021*
Liver cirrhosis	1.080	0.642-1.817	0.771			
Histological differeniation	0.964	0.727-1.278	0.800			
AFP	1.162	0.758-1.781	0.491			
TPD52	0.488	0.312-0.764	0.002*	0.571	0.357-0.911	0.019*

HR, hazard ratio; CI, confidence interval; AFP, alpha-fetoprotein.

* Statistically significant (*P* < 0.05).

**Table 3:** Univariate and multivariate analysis of disease-free survival in HCC

Variables	Univariate analysis			Multivariate analysis		
	HR	95% CI	p value	HR	95% CI	*P* value
Age	1.338	0.866-2.066	0.190			
Gender	1.0.40	0.602-1.794	0.889			
HBV	1.559	0.751-3.237	0.233			
Tumor size	1.137	0.735-1.759	0.564			
Tumor number	1.590	0.941-2.687	0.083			
Tumor encapsulation	1.164	0.879-1.541	0.290			
TNM stage	1.356	0.972-1.356	0.073			
Liver cirrhosis	0.901	0.528-1.536	0.701			
Histological differeniation	1.008	0.756-1.345	0.956			
AFP	1.000	1.000-1.000	0.001*	1.000	1.000-1.000	0.001*
TPD52	0.593	0.379-0.925	0.021*	0.586	0.375-0.916	0.019*

HR, hazard ratio; CI, confidence interval; AFP, alpha-fetoprotein.

* Statistically significant (*P* < 0.05).

### Correlation of TPD52 expression with p21 or apoptosis-related protein in HCC

To understand the mechanisms linking TPD52 expression to hepatocarcinogenesis, we focused our further studies on the association between TPD52 and p21 to investigate whether TPD52 is involved in the regulation of p21 pathway in HCC. Firstly, p21 protein level was detected in the same fresh tissues samples as was use to examine TPD52. Decreased p21 expression was also seen in most tumor tissues (*P* = 0.0024, Figure 2C), and low expression of p21was found in the samples which low express TPD52 (Figure 2A). Moreover, corresponding with TPD52 expression, the expression of p21 was down-regulated in HCC cell lines and was also predominantly decreased in the Hep3B and HepG2 cells (Figure 2D and 2F).

**Figure 5:**
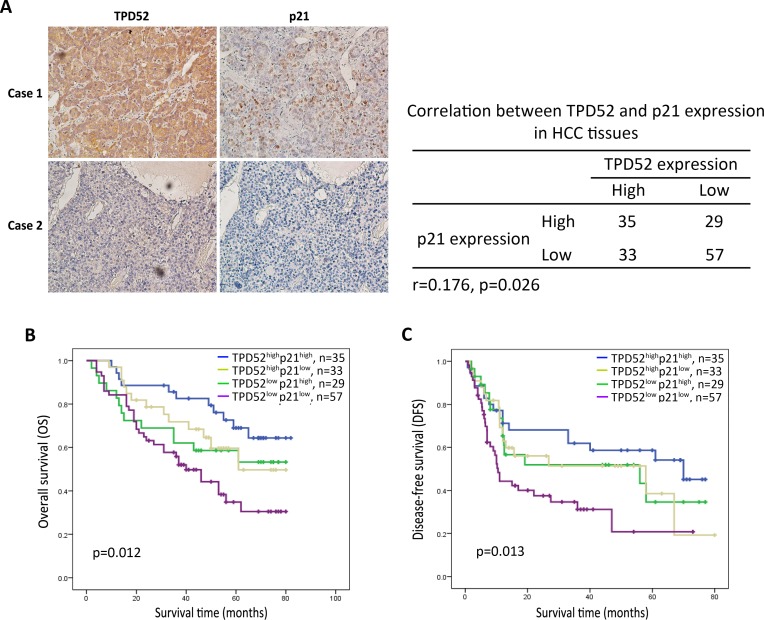
Immunohistochemical analysis for the correlation between TPD52 and p21 protein expression **A.** Immunohistochemical staining of TPD52 or p21 was performed in the serial sections from the same tumor tissues. A summary of the results was shown that there is significant positive correlation between TPD52 and p21 expression in HCC tissues (*p* = 0.026). **B.** and **C.** High expression of both TPD52 and p21 indicated better overall survival and disease-free survival of patients with HCC. (A, ×200 magnification).

In addition, immunohistochemical analysis suggested that p21 expression was positively correlated with TPD52 expression (Figure 5A). All of the 154 patients with HCC were classified into four groups. The TPD52^high^p21^high^ patients were found to exhibit the better overall survival and disease-free survival (*p* = 0.012 and *p* = 0.013, respectively, Figure 5B and 5C). These results indicated that there is a significantly positive correlation between TPD52 and p21, and high TPD52 expression may improve survival of HCC patients through involving in p21 pathway.

**Figure 6:**
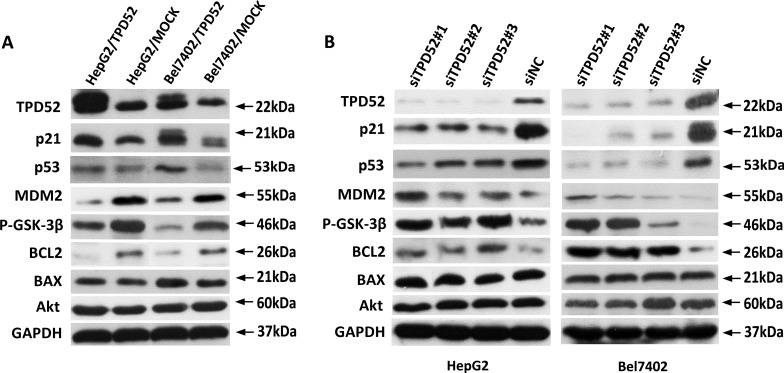
Western blotting detection of apoptosis-related protein **A.** A. Up-regulation of p21 and p53 were detected in TPD52-overexpressed HepG2 and Bel7402 cells. But the expression of MDM2, P-GSK-3β and BCL2 were decreased in TPD52-overexpressed HepG2 and Bel7402 cells. **B.** Down-regulation of p21 and p53 were detected in TPD52-knockdown HepG2 and Bel7402 cells. However, the expression of MDM2, P-GSK-3β and BCL2 were increased in TPD52-knockdown HepG2 and Bel7402 cells.

To further evaluate the molecular mechanism of TPD52 in p21 pathway and the association of TPD52 with apoptotic-related protein in HCC, we generated TPD52-overexpressing cells of which the levels of TPD52 expression were confirmed by western blotting (Figure 6A). The protein levels of p21 and p53 were increased when TPD52 was overexpressed in HCC cells, while MDM2, P-GSK-3β and BCL2 expression were measured strongly decrease in TPD52-overexpressing cells (Figure 6A). Furthermore, we also generated TPD52-knockdown cells. Expectably, TPD52 knockdown was associated with significant down-regulation of p21 and p53 expression and with evident up-regulation of MDM2, P-GSK-3β and BCL2 expression (Figure 6B). However, BAX and Akt were found no significant change in TPD52-knockdown cells or TPD52-overexpressing cells (Figure 6A and 6B).

## DISCUSSION

Currently, a collection of studies on the role of TPD52 in cancers is underway. In our study, we used a relatively large series of clinical tissue samples and cell lines to assess TPD52 expression and its prognostic value in HCC. We measured TPD52 mRNA and protein expression in paired primary HCC tissue samples and HCC cell lines using real-time quantitative PCR and western blotting, respectively. We found that TPD52 expression was down-regulated at both transcriptional and translational level in most primary HCC tumor tissues and HCC cell lines, which was consistent with findings of Tennstedt et al [30]. Immunohistochemical analysis also revealed decreased TPD52 expression in most HCC tumor tissues as compared with the corresponding non-tumor tissues. Correlation analysis showed that decreased TPD52 expression in HCC was significantly associated with TNM stage, suggesting that decreased TPD52 expression may facilitate tumor invasion and infiltration. Kaplan-Meier survival analysis revealed that high TPD52 expression was significantly correlated with favorable prognosis. In addition, multivariate analysis determined that TPD52 expression was an independent prognostic factor for overall survival and disease-free survival. These results suggest that TPD52 might serve as a postoperative prognostic marker for patients with HCC. Corresponding with our study, a recent microarray analysis identified TPD52 over-expression as being associated with improved progression-free survival and overall survival in patients with serous and endometrioid tumors [31]. Another research also found that increased TPD52 expression might be a favorable prognostic marker in ovarian carcinoma [32].

It is generally known that p21 is considered as a potential tumour suppressor via a crucial regulation of cell cycle and senescence in various cancers [33, 34]. Various genetic studies confirmed the ability of p21 to delay tumor development in HCC [35, 36]. In the present study, we found that there is a significantly positive correlation between TPD52 and p21 expression, indicating TPD52 may suppress HCC tumorigenicity and progression through involving in p21 pathway to regulate cell growth and apoptotic. Our further research of apoptotic-related protein showed that p53 expression was also significantly up-regulated or down-regulated in TPD52-overexpressing cells or TPD52-knockdown cells The tumor suppressor p53 is one of the most important cellular gatekeepers for cell cycle arrest, cellular senescence, apoptosis and DNA repair by regulating p21 and other apoptosis-related proteins [37, 38]. The change of P53 affected by TPD52 suggested that TPD52 may play a significant role in cell survival by regulating p53 in p21 pathway. In addition, we found that MDM2, P-GSK-3β and BCL2 were markedly down-regulated in TPD52- overexpressing cells and up-regulated in TPD52-knockdown cells, implying TPD52 might negatively regulate these promoting tumorigenesis factors. Recently, MDM2 was known as the major negative regulator of p53 with multiple inhibitory mechanisms such as preventing the transcriptional coactivator recruitment, inhibiting p53-DNA interaction or p53 indirect translation [39, 40]. Zhang et al. [41] reported that MDM2 can independently reduce p21 stability via proteasome-mediated degradation. Studies on GSK-3 showed that P-GSK-3β (serine 9) is a point of convergence for numerous cell signaling pathways involved in cellular physicological processes, such as cell cycle, differentiation and apoptosis [42, 43]. Recent work demonstrated that GSK-3β is involved in the process of tumorigenesis by participating in the NF-κB-mediated gene transcription, which predicts that GSK-3β would promote cancer cell proliferation [44, 45]. BCL2 is well known as an inhibitor of apoptosis which play an important role in hepatocarcinogenesis [46]. A recent study suggested phosphor-inactivation of GSK-3β (P-GSK-3β) boost the production of BCL2 by enhancing the activity of CREB-ATF-1 in the context of PI3K/Akt activation [47]. Ummanni et al.[48] demonstrated that exogenous TPD52 expression promotes prostate cancer cell migration via ανβ3 integrin by activating the protein kinase B/Akt signaling pathway. However, based on our research, we make our point that TPD52 may restrain HCC tumorigenesis and development via up-regulating the expression of p21 and p53, and down-regulating the expression of tumor promoter including MDM2, P-GSK-3β and BCL2 in the context of PI3K/Akt signaling.

In conclusion, our results confirmed that TPD52 is down-regulated in HCC tissues at both mRNA and protein levels and that low TPD52 expression correlates with poor prognosis in HCC. TPD52 may suppress HCC initiation and development by up-regulating the expression of p21 and p53, and down-regulating the expression of tumor promoter MDM2, P-GSK-3β and BCL2. Our findings suggested that TPD52 may serve as a novel prognostic marker and therapeutic target in HCC, although further research is required to clarify the molecular mechanism of TPD52 in HCC initiation and progression.

## MATERIALS AND METHODS

### Patients and tumor tissue samples

A total of 154 paraffin-embedded samples were collected from HCC patients undergoing hepatectomy at the Sun Yat-sen University Cancer Center (SYSUCC) between 2001 and 2009. All of the patients mentioned above had no received preoperative chemotherapy or radiotherapy. Histological types were assigned according to World Health Organization (WHO) classification criteria. The clinical stage was verified according to the 7th TNM staging system which was updated by The American Joint Committee on Cancer (AJCC)/ International Union Against Cancer (UICC) in 2010 [49]. Another 40 fresh tumor tissues and matched control tissues were obtained from HCC patients who had undergone surgical resection at SYSUCC between 2011 and 2013. Both the cancerous and corresponding noncancerous tissues >2 cm away from the HCC were sampled; the diagnosis was confirmed by pathological examination. The matched fresh tissues were obtained following surgical resection and immediately immersed in RNAlater (Thermo Fisher Scientific, Waltham, MA, USA) to stop RNA degradation, kept at 4°C overnight to ensure thorough penetration of the tissues, then frozen at −80°C until RNA and protein isolation were performed. The investigation was conducted in accordance with the ethical standards and was approved by the SYSUCC Ethics Committee.

### RNA extraction and real-time quantitative PCR

Total RNA was extracted from the 40 pairs fresh tissues using TRIzol (Invitrogen, Carlsbad, CA, USA) according the manufacturer's protocol. The total RNA concentration and quantity were assessed by absorbance at 260 nm using a NanoDrop spectrophotometer (ND-1000; Thermo Fisher Scientific). First-strand complementary DNA (cDNA) synthesis was performed using 1 μg total RNA and M-MLV reverse transcriptase according to the manufacturer's protocol (Promega, Beijing, China). The cDNA was subjected to real-time PCR to evaluate the relative expression levels of *TPD52* and the reference gene glyceraldehyde-3-phosphate dehydrogenase (*GAPDH*). The following primer sequences were used: *TPD52* forward, 5′-GAGGAAGGAGAAGATGTTGC-3′; *TPD52* reverse, 5′-GCCGAATTCAAGACTTCTCC-3′; *GAPDH* forward, 5′-CTCCTCCTGTTCGACAGTCAGC-3′; *GAPDH* reverse, 5′-CCCAATACGACCAAATCCGTT-3′. Each 20-μL reaction volume contained 2 μL cDNA, 0.4 μL each pair of oligonucleotide primer, 7.2 μL nuclease-free water, and 10 μL 2× SYBR Green Master Mix (Promega). The cycling parameters began with 50°C for 2 minutes and 95°C for 2 minutes, and then 40 cycles of amplification at 95°C for 15 seconds, 60°C for 30 seconds, and 72°C for 20 seconds, followed by melting curve analysis. The threshold cycle value (Ct) was measured during the exponential amplification phase, and the amplification plots were analyzed using SDS 2.3 software. The relative expression levels of the target gene *TPD52* were normalized to that of the internal control gene *GAPDH*. The data were analyzed using the comparative threshold cycle (2^−ΔΔCt^) method.

### Protein extraction, western blotting, and analysis

Freshly frozen tissue specimens that also from the 40 pairs fresh tissues, cultured cells (LO2, HepG2, Hep3B, HCCLM6, Bel7402), were lysed in radioimmunoprecipitation assay lysis buffer, and the lysates were cleared by centrifugation (12000 rpm) at 4°C for 30 minutes. Approximately 27 μg protein was run on a 15% sodium dodecyl sulfate-polyacrylamide gel and transferred to a polyvinylidene fluoride membrane. After blocking nonspecific binding sites for 60 minutes with 5% non-fat milk, the membranes were incubated with rabbit monoclonal antibodies against TPD52 (1:1000; Abcam, Cambridge, MA, USA), p21, p53, Akt, P-GSK-3β (serine 9) (1:1000; Cell Signaling Technology, Inc, USA), MDM2, BAX, BCL2 (1:500, Proteintach, Chicago, IL, USA), rabbit monoclonal anti-human antibodies against GAPDH (1:10000; Proteintech, Chicago, IL, USA) overnight at 4°C. Next, the membranes washed three times with Tris-buffered saline with Tween-20 (TBST) for 15 minutes and then incubated with horseradish peroxidase (HRP)-conjugated goat anti-rabbit immunoglobulin G antibody (1:1500; Cell Signaling Technology, Danvers, MA, USA) for 1 hour at room temperature. The membrane was then washed three times with TBST and was developed using an enhanced chemiluminescence system (Cell Signaling Technology). The band intensity was measured by densitometry using Quantity One software (Bio-Rad Laboratories, Hercules, CA, USA).

### Immunohistochemistry

A total of 154 paraffin-embedded tissue blocks were sectioned for immunohistochemistry. The sections were deparaffinized with dimethylbenzene and rehydrated with graded 100%, 95%, 90%, 80%, and 70% ethanol. After three washes in phosphate-buffered saline (PBS) for 3 minutes, the slides were immersed in EDTA (1 mmol/L, pH 8.0) and boiled for 15 minutes in a microwave oven for antigen retrieval. After three rinses in PBS for 3 minutes, 3% hydrogen peroxide was used to block the endogenous peroxidase for 10 minutes at room temperature, and the slides were incubated with primary antibody against TPD52 (1:400;Bioss, Beijing, China) or p21 (1;200;Cell Signaling Technology, Inc, USA) at 4°C overnight in a humidified chamber. After five washes in PBS for 5 minutes, the sections were incubated with HRP-conjugated secondary antibody (Envision™ Detection Kit, GK500705; Gene Tech, Shanghai, China) at room temperature for 30 minutes, and then washed five more times with PBS for 5 minutes. The visualization signal was developed with 3,3′-diaminobenzidine tetrahydrochloride, and the sections were counterstained with 20% hematoxylin. Finally, the slides were dehydrated, cleared, and evaluated. Negative control sections were processed as described above except they were incubated overnight at 4°C in blocking solution without the primary antibody.

The total TPD52 immunostaining score was calculated from the percentage of positively stained tumor cells and the staining intensity. The percent positivity was scored as 0 (<5%, negative), 1 (5%-25%, sporadic), 2 (25%-50%, focal), or 3 (>50%, diffuse). Staining intensity was scored as 0 (no staining), 1 (weak staining), 2 (moderate staining), or 3 (strong staining). The sum score of the immunostaining was calculated as the percentage positive score × staining intensity score, and ranged from 0 to 9. We defined TPD52 expression levels as follows: - (0-1 points), + (2-3 points), ++ (4-6 points), or +++ (>6 points). The sampled patients were divided into low TPD52 expression (TPD52- or TPD52+) or high TPD52 expression groups (TPD52++ or TPD52+++). The p21 immunostaining score was calculated as p21 index (the number of p21 positive cells per 1000 cells counted) and the IHC score was 0 (p21 index <5, no staining), 1 (p21 index 5-50, weak staining), 2 (p21 index 50-100, moderate staining), or 3 (p21 index >100, strong staining). We divided the sample patients into two groups: low p21 expression (0-1 points) or high p21 expression (2-3 points). All immunostained sections were analyzed by two observers who were blinded to the patients’ clinical outcome. There was inter-observer discrepancy in less than 10% of the examined slides, and consensus was reached after further review.

### Cell lines and culture conditions

We obtained the LO2, Bel7402, and HCCLM6 cell lines from the Committee of Type Culture Collection of the Chinese Academy of Sciences (Shanghai, China), and cultured them in RPMI 1640 medium supplemented with 10% heat-inactivated fetal bovine serum (FBS) and 1% penicillin-streptomycin. The HepG2 and Hep3B cell lines obtained from American Type Culture Collection (Manassas, VA, USA) were cultured in Dulbecco's modified Eagle's medium supplemented with 10% heat-inactivated FBS and 1% penicillin-streptomycin. All cells were incubated in a 37°C humidified incubator containing 5% CO_2_.

### RNA oligonucleotides and cell transfections

TPD52 overexpression plasmid (EX-Z3578-M02) and the control clone (EX-EGFP-M02) were obtained from GeneCopoeia (USA). The HepG2 and Bel7402 cells were transfected with the indicated TPD52 plasmid construct using Lipofectamine 2000 according to the manufacturer's instruction.

The small interfering RNAs (siRNAs) for TPD52 knockdown were synthesized by GenePharma (Suzhou, China). The three siRNA sequences were as follows: siTPD52-1#, sense=5′-CCCUGAGGAAGGAGAAGAUTT-3′ and antisense=5′-AUCUUCUCCUUCCUCAGGGTT-3′; siTPD52-2#, sense=5′-GGAAGAGCUAAGAAGAGAATT-3′ and antisense=5′-UUCUCUUCUUAGCUCUUCCTT-3′; siTPD52-3#, sense=5′-GCGGAAACUUGGAAUCAAUTT-3′ and antisense=5′-AUUGAUUCCAAGUUUCCGCTT-3′. The negative control (NC), sense=5′-UUCUCCGAACGUGUCACGUTT-3′ and antisense=5′-ACGUGACACGUUCGGAGAATT-3′. The HepG2 and Bel7402 cells were selected to be transfected with 20 μM siTPD52 or NC for 72 hours using the Lipofectamine RNAi MAX reagent (Invitrogen, USA) according to the manufacturer's protocol.

Both the transfection efficiency were evaluated by western blotting.

### Patient follow-up

Postoperative follow-up was conducted regularly at the outpatient department or follow-up center of Sun Yat-sen University Cancer Center, and included clinical and laboratory examination every 3 months for the first 2 years, every 6 months in the following 3 years, and annually for an additional 5 years or until death, whichever occurred first. Overall survival (OS) and disease-free survival (DFS) were used as a measure of prognosis. OS was defined as the time from surgery to death or the last follow-up. DFS was calculated from the date of surgery to the date of progression, recurrence, death or final follow-up. All follow-up data in this research are available and complete.

### Statistical analysis

Statistical analyses were performed using the Statistical Package for the Social Sciences, version 20.0 (SPSS Inc., Chicago, IL, USA). A paired-samples *t*-test was used to compare TPD52 mRNA and protein expression in HCC tumors with that of their paired adjacent non-tumor tissue samples. The correlation between tumor TPD52 or p21 expression and the clinical and pathological features were analyzed using a chi square test for proportion and Pearson's correlation coefficients. Overall survival curves were calculated using the Kaplan-Meier method and were analyzed with the log-rank test. Cox proportional hazards analysis was used in univariate and multivariate analysis to explore the effects of TPD52 expression and HCC clinicopathological variables on survival. The variables included in multivariate analysis were the one considered statistically significant in univariate analysis. The results were analyzed using the Student *t*-test and are expressed as the mean ± standard deviation. A two-sided P-value < 0.05 was considered statistically significant.
